# HRCM: An Efficient Hybrid Referential Compression Method for Genomic Big Data

**DOI:** 10.1155/2019/3108950

**Published:** 2019-11-16

**Authors:** Haichang Yao, Yimu Ji, Kui Li, Shangdong Liu, Jing He, Ruchuan Wang

**Affiliations:** ^1^School of Computer Science, Nanjing University of Posts and Telecommunications, Nanjing 210023, China; ^2^School of Computer and Software, Nanjing Institute of Industry Technology, Nanjing 210023, China; ^3^Jiangsu High Technology Research Key Laboratory for Wireless Sensor Networks, Nanjing 210023, China; ^4^Institute of High Performance Computing and Big Data, Nanjing University of Posts and Telecommunications, Nanjing 210023, China; ^5^School of Software and Electrical Engineering, Swinburne University of Technology, Melbourne 3122, Australia

## Abstract

With the maturity of genome sequencing technology, huge amounts of sequence reads as well as assembled genomes are generating. With the explosive growth of genomic data, the storage and transmission of genomic data are facing enormous challenges. FASTA, as one of the main storage formats for genome sequences, is widely used in the Gene Bank because it eases sequence analysis and gene research and is easy to be read. Many compression methods for FASTA genome sequences have been proposed, but they still have room for improvement. For example, the compression ratio and speed are not so high and robust enough, and memory consumption is not ideal, etc. Therefore, it is of great significance to improve the efficiency, robustness, and practicability of genomic data compression to reduce the storage and transmission cost of genomic data further and promote the research and development of genomic technology. In this manuscript, a hybrid referential compression method (HRCM) for FASTA genome sequences is proposed. HRCM is a lossless compression method able to compress single sequence as well as large collections of sequences. It is implemented through three stages: sequence information extraction, sequence information matching, and sequence information encoding. A large number of experiments fully evaluated the performance of HRCM. Experimental verification shows that HRCM is superior to the best-known methods in genome batch compression. Moreover, HRCM memory consumption is relatively low and can be deployed on standard PCs.

## 1. Introduction

Since the launch of the International Human Genome Project in 1990, the emergence of high-throughput sequencing technologies, such as single-molecule sequencing technology [1] and next-generation sequencing (NGS) technology [2], has led to the reduction in the cost of genome sequencing and the improvement in the speed of sequencing [3]. Many countries and organizations have launched genomic engineering projects [4–6]. As a variety of sequencing projects unfold, the amount of genomic data generated is exploding, and the growth rate will be faster in the future. By 2025, the genomic data alone will be increased at a rate of 1 zettabase/year (1 Z = 10^21^) [7]. Genomic data is growing faster than storage and transmission bandwidth, putting a lot of pressure on storage and data transmission [8–10]. How to store genomic data efficiently and reduce the pressure of storage and data migration is of great significance in genomic research and application [11].

At present, the genomic data formats mainly include FASTQ, SAM/BAM, VCF, and FASTA, wherein FASTA is a text-based format for representing nucleotide or amino acid sequences [12]. In this format, each base is encoded by a single character and sequence names and annotations are allowed to be added before the sequence. Genome sequences stored in the FASTA format are convenient for researchers to perform gene studies and sequence analysis. Many analysis methods for FASTA genomic data have been proposed, such as sequence alignment [13], sequence comparison [14], sequence factorization [15], and collection of k-mer statistics [16, 17]. So FASTA format is widely used in genomic data storage. At present, there are a large number of FASTA format genomic data; for example, 1000 Genomes Project [5] contains 6.7 TB FASTA genomic data, 1001 Project [4] contains 100 GB FASTA genomic data [18], and more FASTA genomic data will be generated in the future. Therefore, the compression of FASTA format genomic data is very important in genome compression research. In this manuscript, an efficient hybrid referential compression method (HRCM) for FASTA genomic data is proposed. HRCM is a lossless compression method. There exist areas in bioinformatics where lossy compression is required, e.g., for compressing FASTQ quality scores. However, for a FASTA genome file, any loss of information can bring unpredictable consequences to genome analysts. HRCM firstly extracts the information of reference sequence and to-be-compressed sequence. Then, based on the reference sequence ACGT information, the k-mer [19] index is constructed. Based on the index, the longest match searching of the to-be-compressed sequence is made. When more than one sequences are to be compressed, the matched result of the first-level matching will be compressed for the second time. At the same time, the other information of the reference sequence and the to-be-compressed sequence such as lowercase character information matches is searched. Finally, the matched result is encoded and stored.

Compared with the state-of-the-art referential genome compressors, HRCM is more innovative in the following. (i) When more than one sequences are to be compressed, HRCM employs the second-level matching based on the matched results of the first-level matching. Although FRESCO [20] and GDC2 [18] also support the second-level matching, firstly FRESCO is lossy compression, and it is only compatible for {A, C, G, T, N} symbols, and secondly FRESCO needs to select additional sequences as reference sequences for the second-level matching. Moreover, these additional sequences cannot be second-level compressed. HRCM does not need specific additional reference sequences for the second-level matching, which is achieved in the to-be-compressed sequences. GDC2 processes all types of characters at the stage of indexes building and matching, which makes GDC2 very complicated and inefficient. In our experiments, GDC2 could not complete the second-level compression of seven human genomes in 24 hours. (ii) In addition to ACGT sequence stream, HRCM especially compresses the lowercase character information through lowercase character information extraction and matching. Cases of characters represent the repetitive sequence in some sequencing teams and should not be lost in compression. HRCM simultaneously extracts the lowercase character information of the reference sequence and to-be-compressed sequences and conducts matching and assessment of their similarity. The information is encoded and stored according to the matched and assessed result. This method maximizes the similarity between the reference sequence and the to-be-compressed sequence. To the best of our knowledge, this lowercase character information processing method is explicitly described and employed for the first time in referential genome compression. (iii) The hash table creation of HRCM is memory saved. The hash table creation based on k-mer calculates the hash values step by step and avoids converting the whole reference and to-be-compressed sequences into integer sequences, which saves memory consumption. The experiments show the excellent compression and decompression performance of HRCM.

## 2. Related Works

At present, genome sequence files are mostly stored and transmitted by compression, but it is still most common to adopt the general-purpose compression algorithms that are used for the compression of other files, such as PPMD algorithm [21], DEFLATE algorithm [22], and Burrows-Wheeler-Transform (BWT) algorithm [23]. The PPMD algorithm uses the input data stream to predict subsequent symbols to reduce the entropy of the output data. Invented by Phil Katz in 1993, DEFLATE is the cornerstone of most modern compression methods. It combines only two algorithms: preprocessing with LZ77 or LZSS and then encoding with Huffman [24] to get a good compression result quickly. BWT invented in 1994 maximizes the number of consecutive occurrences of the same character by processing a piece of input data reversibly. BWT algorithm itself does not perform any compression; it just simply transforms data which can be more effectively encoded by Run-Length Encoder [25] and other compression algorithms. All of these algorithms are supported by mature tools, such as 7-zip (http://www.7-zip.org) and gzip (http://www.gnu.org/software/gzip/), but none of them is able to achieve a high compression ratio because they do not take the advantage of the characteristics of genome sequences. Special purpose genome compression algorithms proposed in recent years have improved this problem. Special purpose genome compression algorithms, roughly speaking, can be divided into two categories: reference-free compression algorithms and referential compression algorithms.

Reference-free compression algorithms can be divided into three categories further: naive bit encoding algorithms, dictionary-based compression algorithms, and statistical-based compression algorithms. Naive bit encoding algorithms are to express the genome characters with the fixed bit encoding, so that more than one genome character can be stored in one byte [26]. They achieve a limited compression ratio but fast compression speed. Dictionary-based compression algorithms are to find repetitive segments in genome sequences, catalog repetitive segments into dictionaries, and then replace them with dictionary indexes. Lempel-Ziv-based compression algorithm [27], such as LZ77 or LZ78, is a typical example of dictionary-based compression algorithms. It makes use of the repeated sequence in the genome sequence and effectively increases the compression ratio. Statistical-based compression algorithms generate a probabilistic statistical model according to the input data stream, predict the probability of the next character, and use different encoding strategies according to the probability. If the probabilistic statistical model always predicts the next character with a high probability, a good compression ratio can be achieved. The most typical of these algorithms is the XM algorithm [28]. In XM algorithm, the probability distribution of the next character in the genome sequence is estimated by a series of probabilistic estimation models called “expert,” which include Markov expert, context Markov expert, copy expert, and reverse expert. These “expert” predictions synthesize the probability distribution of the next character, followed by the combination with the arithmetic encoder [29]. XM can achieve a high compression ratio, but its compression speed is not ideal. It takes several hours to compress one chromosome of a human genome [30]. In 2012, Mohammed et al. published DELIMINATE method [24], which divides the to-be-compressed sequence file to header data and sequence data and then handles them separately. And lastly, all files generated in the previous processes are further compressed by 7-zip. DELIMINATE achieves better compression ratio than general-purpose compression methods although it uses almost the same compression time. In 2011, 2013, 2016, and 2019, respectively, Pinho et al. published DNAEnc3 [31], MFCompress [32], GeCo [33], and GeCo2 [34] based on Markov models. DNAEnc3 partitions sequence to nonoverlapping blocks of fixed size, which are then encoded by the best one of the Markov models of different orders. MFCompress tool divides the to-be-compressed sequence file into different streams in the preprocessing stage and then adopts multiple competing finite-context models and arithmetic coding to encode different streams according to their different characteristics. GeCo presents an extended finite-context model and shows promising compression results. GeCo2, the improved version of the GeCo tool, enhances the mixture of models, improves the cache-hash approach, and develops the ability to run a context model with inverted repeats only. The performance of these four algorithms is improved in turn. The reference-free compression algorithms can have a compression ratio of 2 to 8 using the internal similarity of the genome sequence, generally higher than that of the general-purpose compression algorithms [35]. But their compression ratio is still limited since they do not take advantage of the similarity between genomes. In pursuit of higher compression ratio, referential genome compression algorithms caught a lot of attention by researchers in recent years.

In 2010, Kuruppu et al. proposed the RLZ [36] algorithm, which converts the to-be-compressed sequence into tuple representation based on the reference sequence. RLZ provides an efficient compression results and supports random access to arbitrary fields of compressed files. RLZ-opt [37], an optimized version of RLZ, improves the compression ratio and decompression speed by improving the genome sequence analysis method. GDC [38] can be seen as an improvement on RLZ-opt. It provides a fast and reliable reference selection algorithm and can choose to compress files based on multiple reference genomes. As a further improvement on GDC, GDC2 [18] compresses large collections of genomes through two phases. For the first phase, the hash table is created according to the reference sequence, and then matching segments are found from the to-be-compressed sequence for compression. For the second phase, the compressed sequence is compressed again using its previous compressed sequences as reference. GDC2 implements the second-level matching as well as multithreaded compression for the first time. In 2012, Pinho et al. published GReEn [39], which draws on the copy model of XM compression method, calculates the probability distribution of base pairs, then creates a hash table, and then encodes according to the probability distribution and the hash table. If the number of failures exceeds the threshold, the k-mer starting point will be redefined to build a new model. iDoComp [40] proposed in 2014 builds indexes for reference sequence based on suffix array. Different from other methods, iDoComp stores the indexes separately in the hard-drive. In the matching stage, iDoComp requires storing matches in memory and then combines the consecutive matches, which are very memory consuming. Both ERGC [41] and NRGC [42] were proposed by Subrata Saha and Sanguthevar Rajasekaran in 2015 and 2016, respectively. They adopt the segmentation matching strategy. The reference sequence and the to-be-compressed sequence are divided into segments with equal length and then matched in each segment, respectively. If not successfully matched, the PPMD algorithm will be adopted directly for compression. iDoComp, ERGC, and NRGC all have the poor robustness and high requirement for the similarity between the reference sequence and the to-be-compressed sequence. If the characteristics of the reference sequence and the to-be-compressed sequence conform to their matching strategy, the compression result will be good; otherwise, the compression result will be poor, and even compression cannot be completed, which can be seen in our experiments. In 2017, Liu et al. proposed HiRGC [43] which employs a global matching strategy named advanced greedy matching. In the preprocessing stage, HiRGC extracts acgt sequence stream and then maps the target acgt sequence stream to the reference sequence stream. Compared with the previous referential compression methods, HiRGC achieves better robustness. But the disadvantage is that the global search may generate inferior matching results and the memory control of HiRGC is not ideal. SCCG [44] carefully combines the segmentation matching used in ERGC/NRGC and the advanced greedy matching used in HiRGC and improves the description efficiency of matched strings. Therefore, the compression ratio is higher than HiRGC, at the cost of using more compression time and more compression/decompression memory usage.

## 3. Materials and Methods

**Figure 1:**
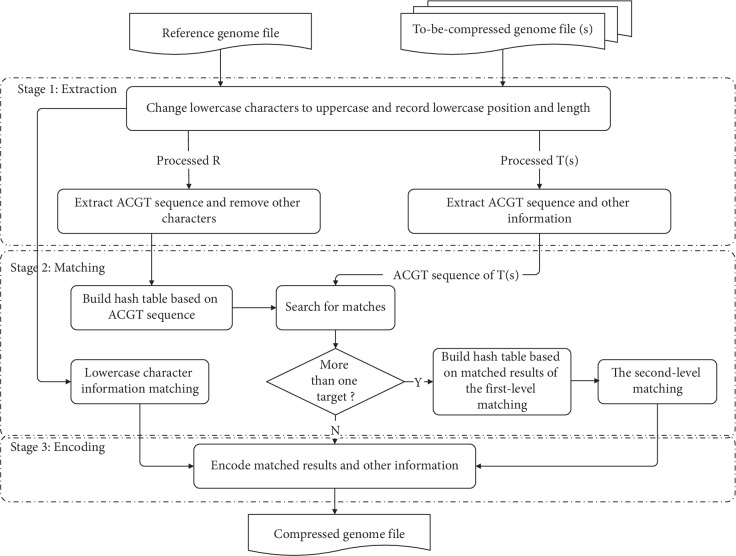
The main process of HRCM.

Compression process of HRCM consists of three stages. (i) *Sequence Information Extraction*. Stage one extracts the information of the reference sequence and the to-be-compressed sequence according to the same dimension. Because HRCM is a lossless compression method, all information of the to-be-compressed sequence must be extracted. (ii) *Sequence Information Matching*. The to-be-compressed information extracted from stage one is matched with the corresponding information of the reference sequence. If more than one sequences are to be compressed, HRCM employs the second-level matching automatically among the to-be-compressed sequences. (iii) *Sequence Information Encoding*. Matched and mismatched information and other to-be-compressed information are encoded. The main process of HRCM is shown in Figure 1. The detailed process of each stage is described in the following sections of this part.  Section 3.4 briefly introduces the decompression of HRCM.

### 3.1. Sequence Information Extraction

Sequence information extraction includes the reference sequence information extraction and the to-be-compressed sequence information extraction; their difference is whether the other information except for ACGT sequence is extracted, that is, identifier stream, N characters, special characters, and line width information. Because the other information in the sequence is stored with little cost, their matching process is not implemented in this version. Therefore, the reference sequence does not need to extract other information except for ACGT sequence but the target sequence does. The steps can be summarized as follows:

- Extract the first line of the sequence as the identifier stream
- Record the character number of one line as the line width of the sequence file
- Change lowercase characters to uppercase and record the lowercase position and length as lowercase information
- Scan the sequence file line-by-line, record the N character position and length as N character information, and record special characters and their positions as special character information
- Delete the identifier stream, line break characters, N characters, and special characters; only reserve ACGT sequence, which is called the basic base sequence B

Their processing algorithms are described in Supplementary Material Algorithms [Supplementary-material supplementary-material-1] and [Supplementary-material supplementary-material-1].

Because, at the encoding stage, the bigger the length and position values are, the more bits are required to represent, the size of the values directly affects the size of the final compressed file. Therefore, we use delta encoding to encode all the *begin* and *position* values. Here is an example as shown in Example 1 to illustrate the first stage of HRCM.

**Example 1 .**

Assume that there are two FASTA format chromosome files X and Y as follows:

  >X_chr1.fa
  AGCTGGGCCCTTaaggNNNnnnXXX
  TTTCCCGGGAAAaaaTTTccctttg
  >Y_chr1.fa
  AGCTGGGCCCTTaaggtttnnnXXX
  TTTCCCGGGNNNaaaTTTccctttg

Assuming that chromosome X is the reference sequence and Y is the to-be-compressed sequence, after the first stage processing, the output of X and Y is as follows:

 Output of chromosome X:
   reference *B* sequence:
   AGCTGGGCCCTTAAGGTTTCCCGGGAAAAAATTTCCCTTTG
   reference lowercase information: {[12, 4], [3, 3], [15, 3], [3, 7]}
 Output of chromosome Y:
   identifier: Y_chr1.fa
   to-be-compressed *B* sequence:
   AGCTGGGCCCTTAAGGTTTTTTCCCGGGAAATTTCCCTTTG
   to-be-compressed lowercase information: {[12, 10], [15, 3], [3, 7]}
   N character information: {[19, 3], [12, 3]}
   other character information: {[22, X], [0, X], [0, X]}
   line width: 25

### 3.2. Sequence Information Matching

At this stage, sequence information matching consists of two parts: one is *B* sequence matching, and the other is lowercase character information matching. *B* sequence matching is based on the reference *B* sequence to find matching segments in the to-be-compressed *B* sequence. If more than one sequences are to be compressed, HRCM employs the second-level matching automatically among the to-be-compressed sequences. The matching strategy of HRCM is selecting the longest match based on separate chaining.

#### 3.2.1. First-Level Matching

In the first step, we create the hash table for reference *B* sequence. This manuscript creates the hash table based on k-mer, that is, k-mer hashing. It is just like a sliding window with slides of length k over the reference sequence and a window containing a k-mer. We digitally encode the A, C, G, T to 0, 1, 2, 3, respectively. In this way, the k-mer is converted into an integer sequence. Then we calculate the hash value of the k-mer. The calculation method assures that different k-mer hash values represent different k-mers. HRCM uses array *H* and array *L* to store the positions of all k-mers. All initial values of *H* are −1. If the hash value of the *i*-th k-mer is expressed as *value*_*i*_, at the stage of hash table creating, *i* will be stored in the value_*i*_-th element of the array *H*, and the original value of the value_*i*_-th element in the array *H* will be stored into the array *L*. The equation can be expressed as *L*[*i*]=*H*[*value*_*i*_], *H*[*value*_*i*_]=*i*. Thus, when an identical k-mer appears in the reference sequence, it will be stored in the array *H* if it is the last one, and otherwise, it will be stored in the array *L*. After calculating a k-mer hashing, the sliding window slides forward one base character until the window cannot slide anymore. Thus, the hash table builds all k-mer indexes of the reference *B* sequence.

The second step of this stage is the longest matching process. First, the value of each k-mer is calculated by the same formula for the to-be-compressed *B* sequence, and then the array *H* is checked to see if the value exists or not. If it does not exist, it means that the k-mer does not exist in the reference sequence, and the base is recorded as a mismatched character; otherwise, it indicates that the k-mer exists, and then searches will traverse all identical k-mers based on the chain constituted by the array *H* and the array *L*. The longest matching segment will be found, the length of the segment is taken as the *length* value, and the position of the segment in the reference *B* sequence is used as the *position* value. The matched segment is represented as a (*position*, *length*) tuple and the to-be-compressed segment is replaced. The algorithm is described as shown in Algorithm 1.

#### 3.2.2. Second-Level Matching

When more than one genome files are to be compressed, HRCM automatically employs batch compression mode. In batch compression mode, the match results of the first-level matching continue to be matched by the second-level matching. The second-level matching is also based on hash matching, but unlike the first-level matching, the elements for matching are not just the four base characters {A, C, G, T} but matched entities (*position*, *length*, *mismatched*). Moreover, there is not just one but multiple reference sequences in the second-level matching, so the search for the longest matching is required to be performed in all reference sequences. Of course, to ensure that the memory usage of compression does not explode with the number of the to-be-compressed sequences, the second-level matching percentage *p*, which means *p*% of the to-be-compressed sequences as the second-level matching reference sequences, can be set. Therefore, the second-level matching is much more complicated.

Firstly, we need to create the hash index for the matched entities output in the first-level matching. In order to ensure as much as possible that different hash values represent different matched entities, we involve the *position*, *length*, and each mismatched nucleotide in the calculation and use large prime number as the multiplier.

For the handling of conflicts, we still employ separate chaining, the same method as the first-level matching. During the search, the to-be-compressed matched entities calculate the hash value in the same way as hash index creating, and the hash tables of each sequence are traversed one by one to find out if the entities with the identical hash value exist. However, unlike the first-level matching, identical hash value does not mean identical entity, because, in the second-level matching, the mapping between matched entity and hash value is not one-to-one mapping. Therefore, when an identical hash value is found, it is necessary to verify if the matched entity is identical. If it is, the search is continued for consecutive matched entities until a different matched entity is found, and then the *sequence id*, *position*, and *length* of the matched segment are recorded. After all the reference sequences have been traversed, the longest matching segment is taken as the final matched segment for replacement and stored as a triple (*sequence_id*, *position*, *length*). For a matched entity, if no identical consecutive matched entity is found after all the reference sequences have been traversed, it is directly stored as the mismatched segment. The matching algorithm of the second-level matching is shown in Algorithm 2.

The processing results of Algorithms 1 and 2 are illustrated by one example shown in Example 2.

**Example 2 .**

Suppose that we are given 6 sequences, with the first sequence as the reference sequence and the last 5 sequences as the to-be-compressed sequences. After the sequence information extraction stage, the 6 output *B* sequences are shown in Table 1. Each *B* sequence contains 20 base characters. In the first-level matching, all the to-be-compressed sequences from T1 to T5 are matched with the reference sequence R1, and the matching result is shown in Table 2. All the to-be-compressed sequences are expressed as triples. In the second-level matching, T2 is matched with T1 as the reference sequence, T3 is matched with T1 and T2 as the reference sequence, T4 is matched with T1, T2, and T3 as the reference sequence, and T5 is matched with T1, T2, T3, and T4 as the reference sequence. The matching result is shown in Table 3, and all the to-be-compressed sequences are mapped to new triples. A comparison of Tables 3 and 1 reveals that the sequence size is greatly reduced.

#### 3.2.3. Lowercase Character Information Matching

All the lowercase character tuples of the to-be-compressed sequences are searched with the reference sequence lowercase character tuples, respectively. If the to-be-compressed lowercase tuple exists in reference lowercase tuple array, it is represented as the location of the same tuple in reference lowercase tuple array, or it is directly stored in a differential lowercase array. For the matching results are continuous in most cases, the run-length-encoding algorithm is improved for the matching results in HRCM, that is, improved from the calculation of the number of continuous same elements to the calculation of the number of elements with continuous tolerance of 1. The storage cost of lowercase character tuples may be greatly reduced by Algorithm 3 and the improved run-length-encoding algorithm.

### 3.3. Sequence Information Encoding

At this stage, the information output at the first and second stages is encoded and stored. The identifier streams and line widths of all the to-be-compressed genome files are encoded through run-length-encoding [25]. Special characters are encoded by static entropy encoding. For most of the *position* value derived from the previous two stages, we employ predictive incremental encoding. Position increases monotonically in most cases, and the main difference between sequences is single nucleotide polymorphisms (SNPs). The *position* in the next matched entity is predicted by the *position* and *length* in the previous matched entity, and the storage space can be reduced by storing the difference between the real *position* and the predicted *position*. Finally, all the above-encoded information is encoded and stored using the PPMD encoder. Currently, there are a lot of tools supporting the PPMD encoder. We use 7-zip (https://www.7-zip.org/) for PPMD encoding and output a 7z-suffixed compressed file.

### 3.4. Decompression

The decompression of the compressed file is the reversion of compression. Firstly, the compressed file is decompressed by 7-zip, then identifier streams and line widths are decompressed by the run-length-encoding algorithm, and special characters are decompressed by the static entropy encoding. At the same time, the reference *B* sequence and lowercase character information are extracted from the reference sequence according to the same method during compression. The complete *B* sequences and lowercase character information of the to-be-compressed files are recovered by combining the recovered matched and mismatched information. Finally, the original genome files are restored. Decompression takes O(D) time and memory for data sets of size D nucleotides and is much less than compression. Therefore, decompression is much faster and more resource-saved than compression.

## 4. Experimental Verification and Analysis

### 4.1. Experimental Environments and Data Sets

In this section, two modes of HRCM method are evaluated. One is the single sequence compression mode (denoted by HRCM-S). In this mode, sequences are compressed one by one. The other is batch compression mode (denoted by HRCM-B). In this mode, all sequences are input together to be compressed by HRCM. All experiments were carried out on machines with 2 × 2.8 GHz Intel Xeon E5-2680 (20 cores) and 32 GB RAM.

The data sets used in our experiments were all retrieved from open access genomic data and widely used in genome compression tests. To evaluate the compression performance of single sequence compression, we picked UCSC sequencing data (hg17, hg18, hg19, hg38), KOREF_20090131 (denoted by K131), KOREF_20090224 (denoted by K224) [45], the first Chinese genome sequence YH [46], and the genome of J. Craig Venter (HuRef) [47] for *Homo sapiens* (denoted by *H. sapiens*) genomic data. Besides, as for other species genomic data, we selected two versions of *Arabidopsis thaliana* (TAIR9, TAIR10), three versions of *Caenorhabditis elegans* (ce6, ce10, ce11), three versions of *Oryza sativa* (TIGR5.0, TIGR6.0, TIGR7.0), and two versions of *Saccharomyces cerevisiae* (sacCer2, sacCer3). To evaluate the compression performance of large collections of genomes, we supplemented two UCSC genomes (hg13, hg16) and 1000 Genome Project [5] for *H. sapiens* genomic data. Each *H. sapiens* genome contains chromosomes 1-22 and XY sex-chromosomes and is about 3 GB in size. The total number of *H. sapiens* genomes is 1102 and the total size is about 3.11TB. In order to verify the compression capability of the HRCM method for only one-version sequences without related reference sequences, we supplemented the DNA sequence corpus published by Pratas et al. [48]. More details of our data sets are shown in the supplementary material data sets section.

### 4.2. Experimental Results and Analysis

In our experiments, we recorded the data of original file size, compressed file size, compression/decompression time, and compression/decompression memory usage and then compared them with those of the six excellent compression methods (iDoComp, GDC2, ERGC, NRGC, HiRGC, and SCCG) published in the last five years. The original file sizes and compressed file sizes only contain the file sizes of the to-be-compressed genomes and do not contain the file sizes of the reference genomes in this manuscript, similar to other related papers.

#### 4.2.1. Compression Results and Analysis of *Homo sapiens* Data Sets

We firstly selected *H. sapiens* for experimentation. Similar to the testing scheme used in HiRGC and SCCG, in our experiments, the same eight genomes of *H. sapiens* were selected and each genome was used in turn as the reference genome to compress other genomes, so as to exclude the contingency caused by the selection of reference and fully evaluate the robustness and practicability of the method. iDoComp and NRGC failed to compress some genome sequences, and we simply compressed the original file with the PPMD compression algorithm as the description in the original papers.

The compressed file sizes of HRCM and the six compared methods together with the corresponding improvement of HRCM-B over other methods are summarized in Table 4. The original file size and compressed file size in the table are the sum of original file sizes and the sum of the compressed file sizes of the 7 to-be-compressed genomes. Relative compression gain quantifies the improvement of HRCM-B compared against other methods, and the calculation method is as follows:

(1) $$\begin{array}{c} \text{gain}=\left(1-\frac{\text{compressed file size by HRCM}-\text{B}}{\text{compressed file size by other method}}\right)\times 100\%. \end{array}$$

As shown in Table 4, HRCM-S compressed about 20 GB of genomic data to 90.89 MB to 152.5 MB. The compression ratio is inferior to SCCG but superior to other methods using hg17, hg18, hg19, and hg38 as the reference sequence. In other groups, the compression ratio of HRCM-S is inferior to HiRGC and SCCG but superior to other methods. However, the batch compression mode HRCM-B compressed these genomic data to 75.38 MB to 97.41 MB and achieved the best result in all groups. The average gain of HRCM-B over HRCM-S is 26.68%. In comparison with other methods, the average gain over GDC2, ERGC, NRGC, HiRGC, and SCCG is 94.57%, 95.65%, 93.99%, 25.08%, and 13.61%, respectively. This means that, storing the same genomic data, other compression methods take up much more disk space than the HRCM method. When the genomic data is huge, the batch compression by HRCM will save more storage and migration expense. The reason why HRCM-B obtains the superiority is because HRCM-B utilizes the similarity of the to-be-compressed sequences through the second-level matching. All detailed compressed sizes are shown in Supplementary Material Tables [Supplementary-material supplementary-material-1] and [Supplementary-material supplementary-material-1].

**Figure 2:**
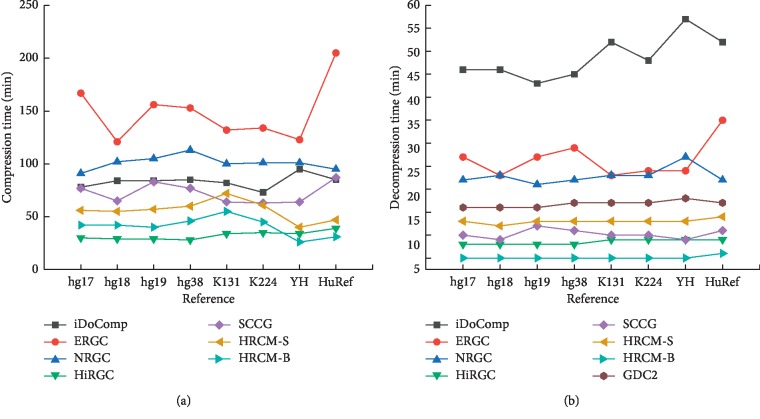
Compression and decompression time of different methods on the eight groups of human genomes. (a) Compression time. (b) Decompression time.

Figure 2 shows the total compression time and decompression time of HRCM and other compared methods on the eight groups of human genomes. Each compression/decompression time in this manuscript was obtained by averaging over three execution times of the same experiment on the same machine. The compression time of GDC2 is not shown in the figure, because it is much more than other methods. In the eight groups of compression experiments, the least compression time of GDC2 was about 10 hours, 13.4 times of HRCM-B. HiRGC achieved 6 best results in the 8 groups of experiments, taken an average of 32 minutes to compress one group of genomes. HRCM-B achieved 2 best results, taken an average of 41 minutes for one group. HRCM-S performed worse than HRCM-B but better than SCCG. In Figure 2(b), we can see that HRCM-B performed the best in all methods, and the decompression was stable and around 5 minutes in all groups.

Next, we continued to use much bigger data sets to evaluate the performance of compressing a large amount of genomic data of HRCM. Besides the above eight human genomes, we supplemented two UCSC genomes (hg13, hg16) and 1000 Genome Project [5] data sets. The total number of *H. sapiens* genomes increased to 1102. We used the hg13 genome as the reference genome to compress the other 1101 genomes. We evaluated the performance of four second-level matching percentages of HRCM-B and compared them with the two optimal algorithms HiRGC and SCCG. The detailed compression results are shown in Supplementary Material Tables [Supplementary-material supplementary-material-1] and [Supplementary-material supplementary-material-1]. In this experiment, the compression ratio of HRCM-B increased without exception as the percentage increased. When the percentage was 5%, HRCM-B consumed 77.34 hours and compressed 3.11TB data to 1756 MB. When the percentage was 20%, the compressed file size was 1379 MB, and the compression time was 84.8 hours. The compression ratios were 1855 and 2362, respectively. HiRGC compressed the data to 23759 MB at the cost of 105 hours. The compressed size by HRCM-B (*p*=20) was only 6% while the compression time was only 75% compared to HiRGC. SCCG failed to compress chromosome 13 and chromosome 14. On other chromosomes, SCCG compressed them to 25043 MB in 270 hours. So on large data sets, HRCM-B increases the advantage as the number of the to-be-compressed genomes increases. Moreover, compression of large collections of genomes is more and more common in storage and migration scenarios. But so far, the available lossless batch compression for FASTA genome files is still lacking. The detailed compression memory of HRCM-B is shown in Supplementary Material [Supplementary-material supplementary-material-1]. The memory consumption of HRCM-B gradually increases with the increase of percentage, but it is still controllable.

#### 4.2.2. Compression Results and Analysis of Other Species Data Sets

In other species data sets experiments, we employed a similar experimental scheme as human genome data sets. Each genome was used in turn as the reference genome to compress other genomes of the same species. The compressed sizes by HRCM and all compared compression methods are shown in Table 5. The compression and decompression time are shown in Supplementary Material Tables [Supplementary-material supplementary-material-1] and [Supplementary-material supplementary-material-1]. All the algorithms have close performance in compression ratio. iDoComp, HRCM, GDC2, and NRGC achieved 4, 3, 2, and 1 best cases, respectively. In compression speed, HRCM-B performed the best. In decompression speed, HRCM-B achieved 7 best cases, which were comparable to HiRGC.

#### 4.2.3. Compression Results and Analysis of Only One-Version Sequences

If a species is only sequenced for one version of genome sequence, which may be the vast majority of the genomic data in the near future, we are interested in whether HRCM can properly compress them and what the compression results are. To our best knowledge, until now there is no research of using referential compression method to compress only one-version genome sequences. We, therefore, selected one version of nonhuman species of our data sets, i.e., ce6, TAIR9, TIGR5.0, and sacCer2. In addition, we selected the DNA sequence corpus proposed recently by Pratas and Pinho [48]. The DNA sequence corpus contains 17 DNA sequences with different sizes and reflects the main domains and kingdoms. HRCM selected BuEb sequence which is a DNA sequence corpus as the reference sequence to compress others and compared the results with widely used general-purpose compression methods gzip, bzip2, lzma, and ppmd and the state-of-the-art special purpose genome compression methods MFCompress [32] and GeCo2 [34]. MFCompress tool was tested in default mode and the best mode (denoted by MFC-2 and MFC-3, respectively, according to the original paper). The detailed compression performance including compressed size and resources requirement (RAM and compression/decompression time) is shown in Supplementary Material Tables [Supplementary-material supplementary-material-1] [Supplementary-material supplementary-material-1]. The experiments verify that HRCM achieves lossless compression on only one-version genomes. The compression ratio is better than widely used general-purpose compression tools in all cases, compared to the specific reference-free compression tools. In the 16 cases of the DNA sequence corpus compression, the most recent compression tool GeCo2 performed the best and achieved all the best cases. HRCM performed slightly inferior and achieved 10 second best cases. MFC-2 and MFC-3 achieved 2 and 4 second best results, respectively. In the 4 real genome data sets, MFCompress performed better than HRCM. GeCo2 is only compatible for {A, C, G, T} symbols and cannot achieve lossless compression.

#### 4.2.4. Memory Usage Analysis

The most consumptive memory of HRCM is the memory consumed in creating the hash table. In HRCM, the hash table length is *L*=2^2*k*^. Each hash entry is allocated 4 bytes memory space, so the memory consumed to the hash table is a *M*=4*L*=2^2(*k*+1)^ byte. Therefore, the value of *k* plays a key role in memory consumption, but meanwhile, the value of *k* also impacts the compression time. Therefore, the selection of *k* is pursuing the balance of compression time and compression memory. To determine the value of *k*, we tuned *k* within the range [11, 15], because if *k* exceeds 15, HRCM occasionally fails with memory allocation on the 32 GB RAM machine. We did the eight benchmark human genomes experiment under different values of *k* and recorded the total compression time and peak memory usage. The results are shown in Supplementary Material Figure 1. As the value of *k* increases, the peak memory usage increases dramatically. However, the compression time does not decrease dramatically as the value of *k* increases. When *k* = 15, the total compression time is almost the same as *k* = 14. But when *k* = 14, the memory consumption is relatively low. HRCM can be run on the cheapest commodity PC. Therefore, we think that *k* = 14 is the best choice.

**Figure 3:**
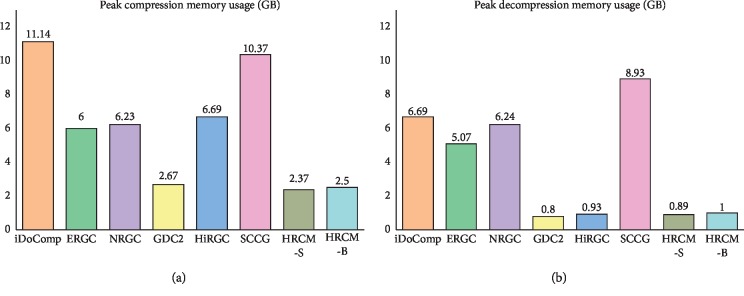
Peak memory usage of different methods. (a) Peak compression memory usage. (b) Peak decompression memory usage.

Figure 3 shows the peak memory usage of all methods on the eight benchmark human genomes experiment. HRCM-S requires the least memory. HRCM-B requires more memory than HRCM-S but still less than other methods. iDoComp requires the most memory. As can be seen from the peak decompression memory usage, GDC2, HiRGC, and HRCM require about 1 GB memory. iDoComp, ERGC, NRGC, and SCCG still have high requirement of memory in decompression.

## 5. Conclusions

In this manuscript, a new lossless compression method for genomic data is proposed and its engineer software is available. The method can perform reference-based as well as reference-free compression with competitive results. When the number of the to-be-compressed sequences is more than one, HRCM improves the compression performance through the second-level matching. The experimental results show that the method has a competitive compression ratio, compression speed, robustness, and memory usage. And the more the number of to-be-compressed sequences, the greater the superiority of HRCM. However, there is still much room for improvement. For the next stage, the first thing is to continue to improve the HRCM's compression ratio. The compression ratio may be improved by analyzing the internal features of sequences and the features of matching results further. The second thing is to realize the distributed processing of the compression algorithm. When a large number of genomes are compressed simultaneously, it is very time-consuming but suitable for distributed processing. The distributed processing of genome compression will greatly improve the compression speed.

## Figures and Tables

**Algorithm 1:**
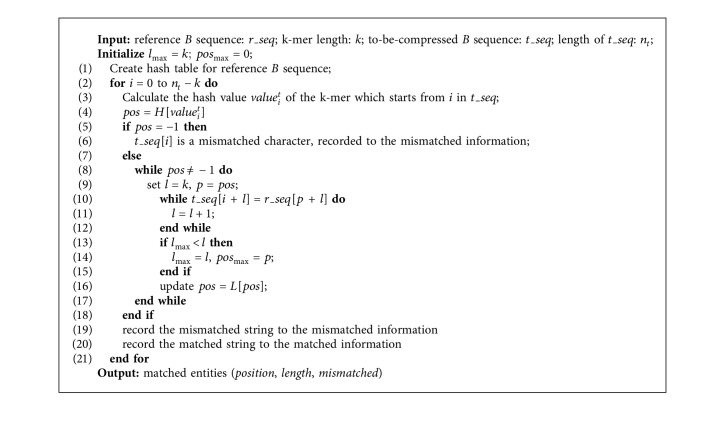
The first-level matching.

**Algorithm 2:**
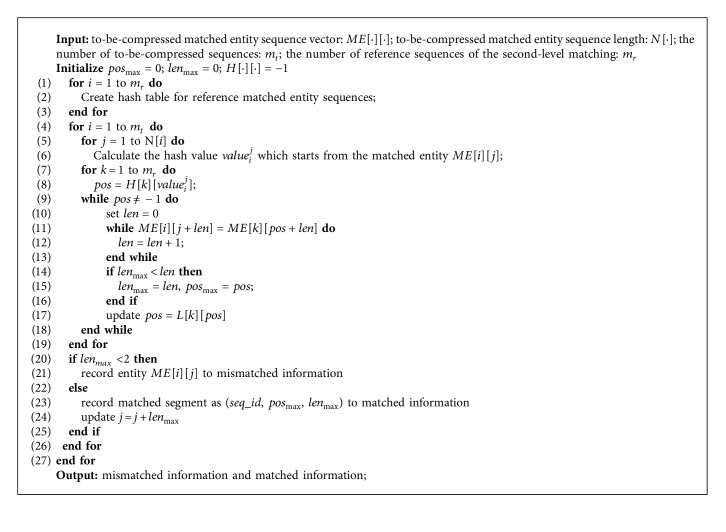
The second-level matching.

**Algorithm 3:**
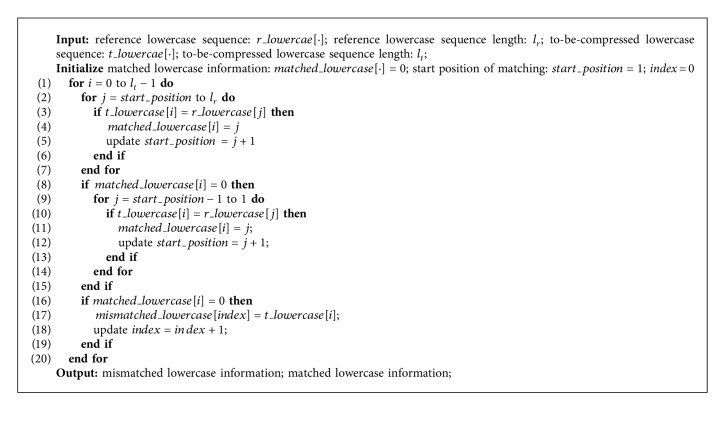
Lowercase character information matching.

**Table 1:** Output *B* sequences after sequence information extraction.

Base sequence	1	2	3	4	5	6	7	8	9	10	11	12	13	14	15	16	17	18	19	20
R1	A	G	A	T	G	G	G	C	C	C	T	T	T	A	G	G	T	A	T	T
T1	A	G	C	T	G	G	T	C	C	C	T	G	A	A	G	G	A	A	T	C
T2	A	G	C	T	G	G	T	C	C	C	T	G	G	A	G	G	A	A	T	C
T3	A	G	T	T	G	G	T	C	C	C	T	G	G	A	G	G	A	T	T	T
T4	A	G	T	T	G	G	T	C	C	C	T	G	A	A	G	G	A	T	T	T
T5	A	T	A	T	G	G	T	C	C	C	T	G	A	A	G	G	A	T	T	T

**Table 2:** Output after the first-level matching.

Base sequence	1	2	3	4	5
T1	(1, 2, C)	(4, 3, T)	(8, 4, GA)	(14, 3, A)	(18, 2, C)
T2	(1, 2, C)	(4, 3, T)	(8, 4, GG)	(14, 3, A)	(18, 2, C)
T3	(1, 2, T)	(4, 3, T)	(8, 4, GG)	(14, 3, AT)	(19, 2)
T4	(1, 2, T)	(4, 3, T)	(8, 4, GA)	(14, 3, AT)	(19, 2)
T5	(1, 1, T)	(3, 4, T)	(8, 4, GA)	(14, 3, AT)	(19, 2)

**Table 3:** Output after the second-level matching.

Base sequence	1	2	3	4
T2	(1, 1, 2)	(8, 4, GG)	(1, 4, 2)	
T3	(1, 2, T)	(2, 2, 2)	(14, 3, AT)	(19, 2)
T4	(3, 1, 2)	(8, 4, GA)	(3, 4, 2)	
T5	(1, 1, T)	(3, 4, T)	(4, 3, 3)	

**Table 4:** Overall comparison of compressed size and the relative gain for different methods under different reference genomes.

Reference	Original file size (MB)	Compressed file size (MB) by							
		iDoComp	GDC2	ERGC	NRGC	HiRGC	SCCG	HRCM-S	HRCM-B
hg17	20,966.28	517.43	1570.94	2220.85	1952.05	103.62	89.09	99.66	**80.13**
		84.51%	94.90%	96.39%	95.90%	22.67%	10.06%	19.60%	
hg18	20,962.74	506.75	1564.89	1498.22	1237.86	97.27	82.05	96.55	**77.89**
		84.63%	95.02%	94.80%	93.71%	19.92%	5.07%	19.32%	
hg19	20,947.88	581.90	1610.37	1826.78	1179.24	95.57	81.56	90.89	**77.43**
		86.69%	95.19%	95.76%	93.43%	18.98%	5.07%	14.81%	
hg38	20,955.10	526.98	1659.51	1708.70	1247.31	96.78	81.93	96.73	**75.38**
		85.70%	95.46%	95.59%	93.96%	22.11%	7.99%	22.07%	
K131	20,972.50	1338.91	1570.45	1874.15	1172.16	124.20	108.53	132.50	**91.80**
		93.14%	94.15%	95.10%	92.17%	26.08%	15.42%	30.71%	
K224	20,972.51	1284.20	1540.93	1897.95	1172.65	124.97	109.44	133.70	**93.56**
		92.71%	93.93%	95.07%	92.02%	25.13%	14.50%	30.02%	
YH	20,972.51	399.29	1643.71	1840.61	1171.03	128.47	113.26	134.50	**93.38**
		76.61%	94.32%	94.93%	92.03%	27.31%	17.56%	30.58%	
HuRef	20,965.32	824.15	1501.46	2911.90	2295.88	146.15	129.33	152.50	**97.41**
		88.18%	93.51%	96.65%	95.76%	33.35%	24.68%	36.13%	

Bold indicates the best value of the case.

**Table 5:** Compressed sizes of other species data sets by different methods.

Reference	To-be-compressed	Original file size (KB)	Compressed file size (KB) by							
			iDoComp	GDC2	ERGC	NRGC	HiRGC	SCCG	HRCM-S	HRCM-B
ce6	ce10, ce11	199,816	251	1049	463	530	445	437	221	**219**
ce10	ce6, ce11	199,804	241	1003	459	641	534	434	**217**	**217**
ce11	ce6, ce10	199,804	463	1941	505	690	489	480	488	**374**
TAIR9	TAIR10	118,360	**2**	3	8	153	5	12	5	5
TAIR10	TAIR9	118,383	**2**	3	5	153	5	2	5	5
TIGR5.0	TIGR6.0, TIGR7.0	740,272	399	**104**	26758	1284	376	363	407	342
TIGR6.0	TIGR5.0, TIGR7.0	740,009	279	**87**	15841	11566	258	257	278	275
TIGR7.0	TIGR5.0, TIGR6.0	739,089	212	79	7091	**66**	227	220	242	168
sacCer2	sacCer3	12,109	**2**	4	6	754	6	3	6	6
sacCer3	sacCer2	12,109	**2**	4	6	517	9	3	6	6

Bold indicates the best value of the case.

## Data Availability

The genome data used to support this study were all retrieved from open access and real genome sequencing data. They are all freely available. The details are shown in Supplementary Material. The source codes of this study are available at https://github.com/haicy/HRCM. They are freely available for noncommercial purposes.

## Supplementary Materials

Supplementary Materials The supplementary material mainly includes the data sets and additional experiments and results that are used to support the conclusions of the manuscript but avoid clustering the main message.
