# Supplementary material for: HRCM: An Efficient Hybrid Referential Compression Method for Genomic Big Data
Source: Biomed Res Int. 2019 Nov 16;2019:3108950. doi: 10.1155/2019/3108950 (PMC6930768; doi:10.1155/2019/3108950)
Supplement: Supplementary Materials — The supplementary material mainly includes the data sets and additional experiments and results that are used to support the conclusions of the manuscript but avoid clustering the main message. [file 3108950.f1.pdf]

HRCM

Supplementary Material

Contents

1 Algorithms 2

2 Data sets...3

2.1 Details of H.sapiens data sets and other species data sets 3

2.2 The 1000 Genomes Project data sets 4

2.3 The DNA sequence corpus 4

3 Additional experiments and results..5

4 References...15

1 Algorithms

Algorithm 1 Reference file information extraction

Input: reference file: r_file, reference file character number: m_r;

Remove the first line of the file;
for $i = 0$ to m_r **do**
  if $r\_file[i]$ = lowercase **then**
    $r\_file[i] = \text{toupper}(r\_file[i])$;
    record the lowercase character position and length as tuple (*position, length*);
  end if
  if $r\_file[i] \in [A, C, G, T]$ **then**
    $r\_seq[j] = r\_file[i]$;
  else
    $r\_file[i]$ is ignored;
  end if
end for

Output: reference ACGT information; reference lowercase character information;

Algorithm 2 To-be-compressed file information extraction

Input: to-be-compressed file: t_file, to-be-compressed file character number: m_t;

Read the first line of the file as *identifier*;
Record the character number of one line as *line_width*;
for $i = 0$ to m_t **do**
  if $t\_file\ [i]$ = lowercase **then**
    $t\_file[i] = \text{toupper}(t\_file[i])$;
    record the lowercase character position and length as tuple (*position, length*);
  end if
  if $t\_file[i] \in [A, C, G, T]$ **then**
    $t\_seq[j] = t\_file[i]$;
  else if $t\_file[i]$ = 'N' **then**
    record the N character position and length as tuple (*position*, *length*);
  else if $t\_file[i]$ = special character **then**
    record the special character information as tuple (*position, character*);
  end if
end for

Output: to-be-compressed ACGT information; to-be-compressed lowercase character information; other information such as *identifier*, *line_width*, N character information and special character information

2 Data sets

2.1 Details of H.sapiens data sets and other species data sets

Table 1: Details of genome data sets used in the experiments

Species	Data sets	Number of Chromosomes	Size	Retrieved from
H.sapiens	hg13	24	2967.55	ftp://hgdownload.soe.ucsc.edu/goldenPath/hg13/chromosomes/
	hg16	24	2986.53	ftp://hgdownload.soe.ucsc.edu/goldenPath/hg16/chromosomes/
	hg17	24	2992.98	ftp://hgdownload.soe.ucsc.edu/goldenPath/hg17/chromosomes/
	hg18	24	2996.52	ftp://hgdownload.soe.ucsc.edu/goldenPath/hg18/chromosomes/
	hg19	24	3011.38	ftp://hgdownload.soe.ucsc.edu/goldenPath/hg19/chromosomes/
	hg38	24	2996.52	ftp://hgdownload.soe.ucsc.edu/goldenPath/hg38/chromosomes/
	K131	24	2986.77	ftp://ftp.kobic.re.kr/pub/KOBIC-KoreanGenome/ KOREF_20090131/fasta/
	K224	24	2986.75	ftp://ftp.kobic.re.kr/pub/KOBIC-KoreanGenome/ KOREF_20090224/fasta/
	YH	24	2986.75	ftp://public.genomics.org.cn/BGI/yanhuang/fa/
	HuRef	24	2993.95	https://www.ncbi.nlm.nih.gov/nuccore Accession:CM000462-CM000485
Arabidopsis thaliana	TAIR9	7	115.62	ftp://ftp.arabidopsis.org/home/tair/Sequences/whole_chromosomes/
	TAIR10	7	115.59	ftp://ftp.arabidopsis.org/home/tair/Sequences/whole_chromosomes/OLD/
Caenorhabditis elegans	ce6	7	97.56	ftp://hgdownload.soe.ucsc.edu/goldenPath/ce6/chromosomes/
	ce10	7	97.57	ftp://hgdownload.soe.ucsc.edu/goldenPath/ce10/chromosomes/
	ce11	7	97.57	ftp://hgdownload.soe.ucsc.edu/goldenPath/ce11/chromosomes/
Oryza sativa	TIGR5.0	12	360.79	http://rice.plantbiology.msu.edu/pub/data/Eukaryotic_Projects/o_sativa/annotation_dbs/pseudomolecules/version_5.0/
	TIGR6.0	12	361.01	http://rice.plantbiology.msu.edu/pub/data/Eukaryotic_Projects/o_sativa/annotation_dbs/pseudomolecules/version_6.0/

	TIGR7.0	12	361.91	http://rice.plantbiology.msu.edu/pub/data/Eukaryotic_Projects/o_sativa/annotation_dbs/pseudomolecules/version_7.0/
Saccharomyces cerevisiae	sacCer2	17	11.86	ftp://hgdownload.soe.ucsc.edu/goldenPath/sacCer2/chromosomes/
	SacCer3	17	11.86	ftp://hgdownload.soe.ucsc.edu/goldenPath/sacCer3/chromosomes/

2.2 The 1000 Genomes Project data sets

The 1000 Genomes Project data sets were retrieved based on the VCF files downloaded from 1000GP FTP server and the script vcf2fasta provided by GDC2 [1].

The reference sequences of assembled chromosomes can be downloaded from:
ftp://ftp.ncbi.nlm.nih.gov/genomes/H_sapiens/Assembled_chromosomes/seq/

The VCF files can be downloaded from:
ftp://ftp.ncbi.nlm.nih.gov/1000genomes/ftp/phase1/analysis_results/integrated_call_sets/

The details of how to use the reference sequences and the VCF files to retrieve the FASTA files are shown in the supplementary material of GDC2.

2.3 The DNA sequence corpus

The data sets can be downloaded from:
https://tinyurl.com/DNAcorpus

More details can refer to the reference [2].

3 Additional experiments and results

Table 2: Detailed compressed sizes by HRCM-S and other methods on the eight benchmark human genomes

Reference	To-be-compressed	Original file size (MB)	Compressed size (MB) by						
			iDoComp	GDC2	ERGC	NRGC	HiRGC	SCCG	HRCM-S
hg17	hg18	2,996.52	**3.37**	11.71	171.85	160.54	10.34	10.30	4.24
	hg19	3,011.38	**8.23**	34.71	379.96	245.32	11.65	11.57	9.32
	hg38	3,004.16	29.59	70.60	580.98	679.55	19.70	**19.46**	20.60
	K131	2,986.77	180.94	388.11	175.11	197.83	18.73	**14.22**	19.60
	K224	2,986.75	118.30	384.77	187.33	179.87	17.29	**12.70**	18.20
	YH	2,986.75	70.25	379.57	202.92	172.17	13.24	**8.66**	14.10
	HuRef	2,993.95	106.75	301.48	522.71	316.76	12.67	**12.18**	13.60
hg18	hg17	2,992.98	**2.26**	10.67	87.62	99.23	9.43	9.42	3.19
	hg19	3,011.38	**6.20**	29.11	299.50	68.70	10.66	10.62	7.56
	hg38	3,004.16	28.11	66.95	582.34	679.56	18.98	**18.74**	19.50
	K131	2,986.77	179.07	389.41	13.51	56.99	17.79	**12.91**	18.50
	K224	2,986.75	116.14	386.09	12.09	56.99	16.34	**11.43**	17.10
	YH	2,986.75	68.64	381.00	7.90	39.54	12.28	**7.60**	18.20
	HuRef	2,993.95	106.34	301.66	495.26	236.85	11.79	**11.33**	12.50
hg19	hg17	2,992.98	**6.17**	33.00	175.75	133.32	9.70	9.68	7.01
	hg18	2,996.52	**5.26**	28.48	131.38	46.64	9.60	9.58	6.28
	hg38	3,004.16	24.14	47.13	526.25	679.38	18.15	17.99	**16.70**
	K131	2,986.77	154.49	402.17	247.19	91.03	18.00	**13.67**	18.80
	K224	2,986.75	193.85	398.87	268.43	43.59	16.56	**12.14**	17.40
	YH	2,986.75	83.82	394.09	190.87	34.04	12.50	**7.94**	13.30
	HuRef	2,993.95	114.16	306.63	286.90	151.25	11.05	**10.57**	11.40
hg38	hg17	2,992.98	11.76	54.57	175.75	201.98	11.09	10.98	**10.90**
	hg18	2,996.52	11.46	52.05	131.38	146.17	11.25	11.14	**10.80**
	hg19	3,011.38	**8.45**	32.82	184.55	144.85	11.55	11.43	9.33
	K131	2,986.77	147.47	407.10	342.37	167.75	19.41	**15.07**	20.40
	K224	2,986.75	167.59	403.77	339.90	208.87	17.96	**13.52**	18.90
	YH	2,986.75	73.55	398.54	266.78	177.23	13.97	**9.37**	15.00
	HuRef	2,993.95	106.71	310.66	267.96	200.48	11.55	**10.42**	11.40
K131	hg17	2,992.98	225.68	335.05	175.75	117.27	19.20	**17.89**	20.00
	hg18	2,996.52	227.07	337.46	131.38	33.42	19.16	**17.44**	19.90
	hg19	3,011.38	353.14	350.60	442.38	106.73	20.43	**19.42**	21.40
	hg38	3,004.16	330.88	370.89	603.92	680.70	28.56	**27.39**	30.40
	K224	2,986.75	6.91	16.18	4.73	7.86	8.88	**4.45**	10.80
	YH	2,986.75	48.72	40.82	8.78	23.04	13.09	**7.82**	13.80
	HuRef	2,993.95	146.52	119.45	507.21	203.14	14.88	**14.12**	16.20

	hg17	2,992.98	232.61	331.57	175.75	117.24	19.12	**17.81**	19.90
	hg18	2,996.52	234.13	333.97	131.38	33.37	19.08	**17.36**	19.80
	hg19	3,011.38	321.95	347.21	443.20	106.78	20.35	**19.34**	21.30
K224	hg38	3,004.16	329.49	367.53	609.98	680.77	28.48	**27.34**	30.30
	K131	2,986.77	6.66	13.19	6.03	8.86	10.19	**5.84**	12.40
	YH	2,986.75	28.68	34.49	8.68	22.60	12.97	**7.72**	14.00
	HuRef	2,993.95	130.68	112.96	522.93	203.03	14.77	**14.03**	16.00
	hg17	2,992.98	47.48	338.22	175.75	114.96	17.71	**16.65**	18.30
	hg18	2,996.52	47.45	340.66	131.38	30.82	17.63	**16.34**	18.20
	hg19	3,011.38	76.09	353.88	433.45	84.78	18.93	**18.10**	19.70
YH	hg38	3,004.16	99.58	434.66	580.58	680.34	27.13	**26.11**	28.80
	K131	2,986.77	19.19	42.58	12.63	28.88	17.13	**12.00**	17.60
	K224	2,986.75	46.00	38.24	11.21	27.44	15.64	**10.50**	16.50
	HuRef	2,993.95	63.51	95.47	495.61	203.82	14.29	**13.57**	15.40
	hg17	2,992.98	98.38	274.07	175.75	303.90	19.97	**19.17**	20.60
	hg18	2,996.52	121.08	275.38	131.38	251.23	20.02	**19.20**	20.60
	hg19	3,011.38	152.13	280.34	464.86	231.16	20.31	**19.56**	20.70
HuRef	hg38	3,004.16	230.50	300.76	586.02	673.91	26.62	**25.82**	28.00
	K131	2,986.77	83.87	135.09	519.18	301.08	21.84	**17.42**	22.90
	K224	2,986.75	81.38	130.05	503.13	287.62	20.34	**15.84**	21.40
	YH	2,986.75	56.80	105.78	531.57	246.98	17.05	**12.32**	18.30

Bold indicates the best value of the case.

Table 3: Detailed compressed sizes of each chromosome by HRCM-B on the eight benchmark human genomes

Chromosome	Compressed size (MB) of each chromosome on different reference genomes							
Number	hg17	hg18	hg19	hg38	K131	K224	YH	HuRef
Chr1	7.71	7.15	7.07	7.25	8.41	8.50	8.43	7.39
Chr2	5.71	5.57	5.49	5.70	6.72	6.80	6.86	7.40
Chr3	4.74	4.66	4.68	4.61	5.65	5.72	5.76	6.20
Chr4	4.80	4.69	4.58	4.75	5.62	5.69	5.72	6.07
Chr5	4.26	4.21	4.25	4.10	5.09	5.16	5.16	5.65
Chr6	4.91	4.85	4.76	4.74	5.72	5.78	5.82	5.21
Chr7	4.21	4.10	4.07	4.03	4.55	4.98	4.55	5.18
Chr8	3.39	3.35	3.35	3.52	4.09	4.14	4.19	4.62
Chr9	3.68	3.47	3.49	3.36	4.05	4.08	4.13	4.66
Chr10	3.81	3.77	3.81	3.74	4.41	4.45	4.44	4.84
Chr11	3.38	3.35	3.39	3.43	3.98	4.03	4.08	4.43
Chr12	3.28	3.22	3.18	3.23	3.93	3.98	4.00	4.36
Chr13	2.56	2.53	2.55	2.44	2.96	3.00	3.02	3.23
Chr14	2.28	2.26	2.28	2.20	2.67	2.71	2.71	2.94
Chr15	2.22	2.20	2.20	2.19	2.58	2.61	2.61	2.82
Chr16	2.47	2.45	2.48	2.39	2.86	2.89	2.93	2.96

Chr17	2.23	2.22	2.23	1.99	2.64	2.68	2.68	2.81
Chr18	1.94	1.93	1.90	1.83	2.23	2.26	2.27	2.41
Chr19	1.66	1.65	1.66	1.55	1.96	1.99	2.02	2.16
Chr20	2.21	2.19	2.18	1.69	2.57	2.59	2.58	2.37
Chr21	2.05	2.05	1.80	1.18	2.20	2.22	2.21	2.01
Chr22	1.86	1.80	1.79	1.17	2.01	2.03	2.01	2.11
ChrX	3.78	3.50	3.53	3.66	4.14	4.47	4.36	4.89
ChrY	1.00	0.71	0.71	0.64	0.77	0.80	0.84	0.71
Total	80.13	77.89	77.43	75.38	91.80	93.56	93.38	97.41

Table 4: Detailed compressed sizes of large collections of genomes by HRCM and other compared methods

To-be-compressed chromosome number	Original file size (MB)	Compressed size (MB) by					
		HiRGC	SCCG	HRCM-B (*p*=5)	HRCM-B (*p*=10)	HRCM-B (*p*=15)	HRCM-B (*p*=20)
chr1	265,231.77	2,475.09	2,431.08	141.28	127.36	122.03	**112.33**
chr2	258,893.91	2,365.94	2,327.49	148.24	132.29	126.24	**116.25**
chr3	210,796.86	430.66	2,861.39	112.85	101.40	97.35	**89.56**
chr4	203,284.71	1,272.56	1,245.77	120.90	107.74	102.76	**93.76**
chr5	192,592.79	1,187.50	1,162.34	107.26	96.74	92.70	**85.46**
chr6	182,102.09	619.34	600.46	103.95	92.19	87.86	**80.71**
chr7	169,370.15	892.30	873.08	96.60	86.65	82.84	**76.04**
chr8	155,750.48	1,605.39	1,578.74	98.20	88.40	84.73	**78.23**
chr9	150,259.34	1,001.40	978.79	71.72	64.72	61.68	**56.66**
chr10	144,293.59	541.09	525.80	79.13	71.02	67.85	**62.09**
chr11	143,680.56	1,070.46	1,047.88	80.84	73.23	70.22	**64.58**
chr12	142,399.72	2,651.88	2,619.23	94.20	84.15	80.15	**72.46**
chr13	122,600.09	333.95	/	53.60	48.30	46.13	**42.65**
chr14	114,222.28	390.53	/	50.66	45.46	43.63	**40.13**
chr15	109,143.69	767.50	752.53	49.92	45.33	43.56	**39.74**
chr16	96,191.27	1,211.78	1,191.28	57.65	51.92	49.79	**46.13**
chr17	86,397.23	1,784.34	1,762.98	56.45	51.15	48.94	**45.33**
chr18	83,120.90	401.86	391.79	43.95	39.89	38.18	**35.28**
chr19	62,903.20	222.05	211.29	37.48	34.13	32.62	**30.22**
chr20	67,082.51	164.38	157.81	33.93	31.30	30.25	**28.06**
chr21	51,209.90	420.28	412.37	22.87	20.85	19.98	**18.66**
chr22	54,541.28	296.98	289.43	23.53	21.57	20.69	**19.23**
chrX	163,753.35	1,567.86	1,539.11	68.90	63.55	56.47	**43.66**

chrY	29,133.14	84.80	82.57	2.16	2.16	2.15	**2.14**
Total	3,258,954.79	23,759.93	25,043.19?	1,756.25	1,581.49	1,508.81	**1,379.36**

" /" indicates that the method failed to compress this chromosome.

"?" indicates that the value does not contain the sizes of the failed chromosomes.

The value of *p* means the percentage of the second-level matching.

Bold indicates the best value of the case.

Table 5: Detailed compression time of large collections of genomes by HRCM and other compared methods

To-be-compressed chromosome number	Original file size (MB)	Compression time (hour) by					
		HiRGC	SCCG	HRCM-B (p=5)	HRCM-B (p=10)	HRCM-B (p=15)	HRCM-B (p=20)
chr1	265,231.77	11.95	21.25	**11.19**	12.23	11.86	11.64
chr2	258,893.91	10.30	19.80	**7.77**	8.70	8.50	8.95
chr3	210,796.86	5.96	19.48	4.37	4.75	**3.96**	5.21
chr4	203,284.71	5.11	13.74	**4.57**	4.94	4.72	5.00
chr5	192,592.79	5.51	16.11	**3.86**	4.63	4.27	4.64
chr6	182,102.09	4.52	13.74	**3.24**	3.85	3.78	4.20
chr7	169,370.15	4.85	12.40	4.21	**3.84**	3.86	3.94
chr8	155,750.48	4.40	11.15	**3.47**	3.59	3.89	3.91
chr9	150,259.34	3.60	12.00	**2.57**	3.27	3.10	3.05
chr10	144,293.59	3.48	9.35	**2.59**	2.77	2.83	2.85
chr11	143,680.56	3.74	12.45	**2.68**	3.03	3.03	3.22
chr12	142,399.72	6.92	12.74	**6.22**	6.54	6.32	6.61
chr13	122,600.09	2.67	/	**1.64**	**1.62**	1.80	1.77
chr14	114,222.28	2.61	/	**1.59**	1.71	1.75	1.76
chr15	109,143.69	3.31	10.44	**1.92**	2.04	2.04	2.04
chr16	96,191.27	4.43	7.41	**2.63**	2.77	2.92	2.78
chr17	86,397.23	5.46	9.95	4.33	4.49	4.50	**4.24**
chr18	83,120.90	2.56	7.46	**1.11**	1.17	1.17	1.21
chr19	62,903.20	2.86	5.44	**1.51**	1.64	1.56	1.59
chr20	67,082.51	2.19	4.77	**0.87**	0.90	0.93	0.94
chr21	51,209.90	1.35	16.95	**0.59**	0.61	0.61	0.64
chr22	54,541.28	1.54	16.78	**0.61**	0.64	0.66	0.67
chrX	163,753.35	5.17	14.21	3.52	**2.83**	3.70	3.66
chrY	29,133.14	0.65	2.36	0.27	0.27	**0.26**	0.27
Total	3,258,954.7	105.14	269.96?	**77.34**	82.84	82.01	84.80

" /" indicates that the method failed to compress this chromosome.

"?" indicates that the value does not contain the compression time of the failed chromosomes.

The value of *p* means the percentage of the second-level matching.

Bold indicates the best value of the case.

Table 6: Detailed compression memory usage of large collections of genomes by HRCM-B

To-be-compressed chromosome number	Compression memory (GB) by			
	HRCM-B (*p*=5)	HRCM-B (*p* =10)	HRCM-B (*p* =15)	HRCM-B (*p* =20)
chr1	4.60	6.90	9.23	11.55
chr2	4.60	6.84	9.10	11.37
chr3	2.94	3.78	4.62	5.47
chr4	3.49	4.92	6.35	7.80
chr5	3.36	4.67	6.00	7.31
chr6	2.87	3.79	4.72	5.65
chr7	2.97	4.07	5.15	6.27
chr8	3.37	4.93	6.48	8.02
chr9	2.83	3.97	5.12	6.27
chr10	2.59	3.41	4.24	5.05
chr11	2.94	4.12	5.30	6.47
chr12	3.93	6.12	8.30	10.49
chr13	2.18	2.81	3.44	4.06
chr14	2.15	2.79	3.42	4.05
chr15	2.36	3.23	4.09	4.95
chr16	2.63	3.77	4.92	6.06
chr17	2.96	4.41	5.87	7.31
chr18	2.07	2.68	3.30	3.92
chr19	2.07	2.24	2.69	3.14
chr20	1.78	2.20	2.60	3.02
chr21	1.76	2.30	2.82	3.36
chr22	1.67	2.12	2.57	3.02
chrX	3.34	4.81	6.28	7.76
chrY	1.35	1.51	1.66	1.81
Maximum	4.60	6.90	9.23	11.55

The value of *p* means the percentage of the second-level matching.

Maximum means the peak memory usage of this method when compressing the data sets.

Table 7: Compression time of other species data sets by different methods

Reference	To-be-compressed	Compression time (s) by							
		iDoComp	GDC2	ERGC	NRGC	HiRGC	SCCG	HRCM-S	HRCM-B
ce6	ce10,ce11	28	38	41	100	37	35	21	**15**
ce10	ce6,ce11	26	22	40	66	29	31	19	**13**
ce11	ce6,ce10	28	29	458	66	30	30	24	**14**
TAIR9	TAIR10	37	18	21	37	15	16	**13**	**13**

TAIR10	TAIR9	32	18	48	37	14	17	**13**	**13**
TIGR5.0	TIGR6.0,TIGR7.0	133	140	156	165	66	202	66	**42**
TIGR6.0	TIGR5.0,TIGR7.0	115	106	107	151	67	143	72	**43**
TIGR7.0	TIGR5.0,TIGR6.0	131	112	238	153	67	151	73	**42**
sacCer2	sacCer3	4	6	8	7	37	**2**	9	9
sacCer3	sacCer2	4	6	7	8	39	**2**	9	9

Bold indicates the best value of the case.

Table 8: Decompression time of other species data sets by different methods

Reference	To-be-compressed	Decompression time (s) by							
		iDoComp	GDC2	ERGC	NRGC	HiRGC	SCCG	HRCM-S	HRCM-B
ce6	ce10,ce11	6	13	8	11	5	7	8	**4**
ce10	ce6,ce11	8	13	9	10	**5**	7	7	**5**
ce11	ce6,ce10	8	12	10	11	**5**	6	7	**5**
TAIR9	TAIR10	**3**	/	4	/	4	3	5	5
TAIR10	TAIR9	**4**	/	5	/	**4**	**4**	**4**	**4**
TIGR5.0	TIGR6.0,TIGR7.0	25	29	16	30	16	24	24	**15**
TIGR6.0	TIGR5.0,TIGR7.0	22	32	18	26	**13**	21	24	17
TIGR7.0	TIGR5.0,TIGR6.0	22	31	18	24	**13**	22	24	21
sacCer2	sacCer3	**1**	4	**1**	5	**1**	**1**	**1**	**1**
sacCer3	sacCer2	**1**	5	**1**	6	**1**	**1**	**1**	**1**

Bold indicates the best value of the case.

"/" indicates that the method failed to decompress the file.

Table 9: Compressed sizes of HRCM and other reference-free methods in reference-free compression experiment

To-be-compressed	Original file size (B)	Compressed file size (B) by							
		gzip	bzip2	lzma	ppmd	MFC-2	MFC-3	GeCo2	HRCM-S
AeCa	1,591,049	498,251	463,727	443,908	419,575	410,373	410,947	**380,115**	388,949
AgPh	43,970	14,090	13,061	13,108	12,664	11,762	11,860	**10,708**	11,991
AnCa	142,189,675	44,021,249	40,794,149	34,870,625	36,871,748	33,573,162	33,098,570	**29,481,172**	34,257,156
DaRe	62,565,020	19,043,617	17,727,572	14,832,647	16,025,115	14,725,780	14,548,815	**11,488,819**	14,846,130
DrMe	32,181,429	10,049,893	9,420,981	8,695,711	8,461,425	8,282,675	8,242,863	**7,481,093**	7,864,880
EnIn	26,403,087	8,252,413	7,696,896	6,717,422	6,864,989	6,471,750	6,381,059	**5,170,889**	6,381,047
EsCo	4,641,652	1,457,983	1,356,790	1,277,986	1,226,095	1,196,397	1,198,507	**1,098,552**	1,139,218
GaGa	148,532,294	46,016,176	42,973,370	39,516,291	38,396,666	37,376,513	37,215,495	**33,877,671**	35,674,338
HaHi	3,890,005	1,208,806	1,120,129	1,060,983	999,799	981,901	981,987	**902,831**	926,283
HePy	1,667,825	513,864	478,440	448,682	425,062	415,195	415,328	**375,481**	392,920
HoSa	189,752,667	58,071,457	53,472,930	47,285,849	48,256,912	45,070,974	44,585,616	**38,845,642**	44,705,871
OrSa	43,262,523	13,442,311	12,618,218	10,740,878	11,391,627	10,541,817	10,428,913	**8,646,543**	10,590,219
PlFa	8,986,712	2,730,066	2,565,298	2,291,492	2,262,496	2,166,310	2,162,922	**1,925,726**	2,095,075
ScPo	10,652,155	3,358,134	3,128,567	2,922,245	2,823,232	2,735,848	2,738,035	**2,518,963**	2,627,654
WaMe	9,144,432	2,888,155	2,685,749	2,542,882	2,434,559	2,383,832	2,387,012	**2,217,655**	2,266,185

YeMi	73,689	22,806	21,184	20,777	19,721	18,373	18,454	**16,798**	18,388
ce6	102,293,252	31,814,131	28,900,571	26,789,336	25,669,209	22,719,271	**22,644,005**	20,028,845*	23,980,249
TAIR9	121,223,713	35,967,576	33,743,432	30,340,560	30,296,845	27,525,512	**27,389,078**	27,254,298*	28,780,372
TIGR5.0	378,279,319	113,872,933	107,018,536	89,043,109	96,932,020	77,762,707	**76,625,018**	77,231,172*	91,017,382
sacCer2	12,399,942	3,822,046	3,568,860	3,431,368	3,209,679	**2,904,003**	2,910,370	2,883,018*	3,047,403

Bold indicates the best value of the case. Gray indicates the second best value of the case.

* indicates GeCo2 cannot achieve lossless compression in these data sets because it is only compatible for {A, C, G, T} symbols.

Table 10: Compression time of HRCM and other reference-free methods in reference-free compression experiment

To-be-compressed	Original file size (B)	Compression time (s) by							
		gzip	bzip2	lzma	ppmd	MFC-2	MFC-3	GeCo2	HRCM-S
AeCa	1,591,049	0.37	0.17	0.93	**0.13**	0.63	1.52	0.72	0.81
AgPh	43,970	0.21	0.14	0.37	0.17	0.20	0.56	**0.01**	0.38
AnCa	142,189,675	27.22	12.72	127.32	**6.86**	39.27	44.11	55.53	22.27
DaRe	62,565,020	11.83	5.73	62.59	**3.47**	16.93	19.33	67.49	9.77
DrMe	32,181,429	6.33	2.92	26.66	**1.63**	6.68	10.74	34.37	5.44
EnIn	26,403,087	5.30	2.39	27.41	**1.39**	6.47	8.72	27.40	4.54
EsCo	4,641,652	0.97	0.46	3.19	**0.32**	1.19	1.97	1.90	1.16
GaGa	148,532,294	28.53	13.12	150.32	**7.14**	31.84	46.33	160.81	23.76
HaHi	3,890,005	0.83	0.41	2.49	**0.25**	1.26	1.72	1.60	1.02
HePy	1,667,825	0.39	0.20	1.10	**0.12**	0.65	1.43	0.77	0.63
HoSa	189,752,667	35.87	17.19	185.80	**8.90**	51.43	58.47	225.27	29.36
OrSa	43,262,523	8.41	3.90	36.24	**2.15**	12.13	13.93	46.23	7.06
PlFa	8,986,712	1.81	0.85	6.77	**0.49**	2.61	3.22	10.44	1.79
ScPo	10,652,155	2.21	1.70	8.36	**0.62**	3.21	3.85	11.06	2.11
WaMe	9,144,432	1.93	0.89	6.68	**0.53**	2.77	3.40	3.68	1.87
YeMi	73,689	0.30	0.20	0.57	0.17	0.21	0.56	**0.05**	0.37
ce6	102,293,252	22.61	9.82	95.61	**6.50**	31.36	46.94	47.48	22.96
TAIR9	121,223,713	23.20	12.32	122.35	**7.19**	35.62	61.39	62.46	23.52
TIGR5.0	378,279,319	71.16	34.79	375.77	**19.37**	100.72	151.80	193.60	71.89
sacCer2	12,399,942	2.89	1.36	6.54	**19.37**	7.67	18.22	8.99	14.47

Bold indicates the best value of the case.

Table 11: Decompression time of HRCM and other reference-free methods in reference-free compression experiment

Sequence	Decompression time (s) by							
	gzip	bzip2	lzma	ppmd	MFC-2	MFC-3	GeCo2	HRCM-S
AeCa	0.21	0.91	0.33	**0.12**	0.57	0.87	0.74	0.13
AgPh	0.70	0.80	0.60	0.17	0.19	0.55	0.02	**0.02**
AnCa	**1.62**	5.61	2.43	7.23	29.33	30.90	55.68	7.33
DaRe	**0.48**	2.45	0.92	3.18	11.53	14.20	66.89	3.43
DrMe	**0.28**	1.33	0.50	1.70	5.53	7.28	34.23	1.87

EnIn	**0.25**	1.94	0.42	1.41	5.94	6.35	27.43	1.51
EsCo	0.57	**0.25**	0.91	0.31	1.86	1.48	1.96	0.34
GaGa	**1.10**	5.86	2.00	7.56	27.16	30.43	161.69	8.33
HaHi	0.46	**0.20**	0.77	0.27	0.89	1.91	1.61	0.27
HePy	0.25	**0.10**	0.44	0.13	0.45	0.88	0.75	0.13
HoSa	**1.37**	7.38	2.58	9.51	38.15	39.66	224.18	10.53
OrSa	**0.38**	1.86	0.66	2.26	8.70	9.93	45.90	2.41
PlFa	0.97	0.43	**0.16**	0.53	1.74	2.38	10.46	0.57
ScPo	**0.11**	0.49	0.19	0.68	2.28	2.78	11.12	0.65
WaMe	0.96	0.42	**0.17**	0.55	1.89	2.49	3.72	0.56
YeMi	0.80	**0.11**	0.80	0.21	0.20	0.54	0.06	0.01
ce6	**0.90**	5.18	1.80	6.64	23.47	36.55	41.41	6.34
TAIR9	**1.61**	5.71	2.14	7.32	27.96	37.77	66.43	7.55
TIGR5.0	**3.29**	16.47	6.90	21.50	92.16	104.18	183.12	21.94
sacCer2	**0.12**	0.65	0.25	0.82	5.21	13.26	8.64	1.63

Bold indicates the best value of the case.

Table 12: Compression memory usage of HRCM and other reference-free methods in reference-free compression experiment

To-be-compressed	Compression memory (KB) by							
	gzip	bzip2	lzma	ppmd	MFC-2	MFC-3	GeCo2	HRCM-S
AeCa	**700**	7,000	96,120	5,660	526,680	2,377,732	526,048	554,584
AgPh	**700**	7,000	96,120	5,660	526,680	2,377,732	?	1,053,980
AnCa	**700**	7,000	96,120	5,660	526,680	2,377,732	526,120	1,350,360
DaRe	**700**	7,000	96,120	5,660	526,680	2,377,732	2,428,728	1,184,492
DrMe	**700**	7,000	96,120	5,660	526,680	2,377,732	2,428,728	1,118,568
EnIn	**700**	7,000	96,120	5,660	526,680	2,377,732	2,394,160	1,105,636
EsCo	**700**	7,000	96,120	5,660	526,680	2,377,732	526,120	1,053,116
GaGa	**700**	7,000	96,120	5,660	526,680	2,377,732	3,442,748	1,371,920
HaHi	**700**	7,000	96,120	5,660	526,680	2,377,732	526,116	1,055,596
HePy	**700**	7,000	96,120	5,660	526,680	2,377,732	526,116	939,360
HoSa	**700**	8,000	96,120	5,660	526,680	2,377,732	4,001,596	1,452,340
OrSa	**700**	7,000	96,120	5,660	526,680	2,377,732	2,428,728	1,142,376
PlFa	**700**	7,000	96,120	5,660	526,680	2,377,732	1,870,396	1,067,220
ScPo	**700**	7,000	96,120	5,660	526,680	2,377,732	1,869,872	1,073,730
WaMe	**700**	7,000	96,120	5,660	526,680	2,377,732	526,048	1,068,724
YeMi	**700**	7,000	96,120	5,660	526,680	2,377,732	?	1,050,048
ce6	**816**	7,664	96,120	10,484	526,716	2,377,756	526,124	1,096,080
TAIR9	**812**	7,180	96,120	5,736	536,848	2,392,360	526,124	1,109,032
TIGR5.0	**816**	7,904	96,120	5,752	541,116	2,377,744	526,124	1,143,528
sacCer2	**820**	7,028	32,328	7,768	526,700	2,377,768	526,124	1,054,368
Maximum	**820**	7,904	96,120	10,484	541,116	2,392,360	4,001,596	1,452,340

Bold indicates the best value of the case.

"?" indicates that the compression is too fast to record the memory usage.

Maximum means the peak memory usage of this method when compressing the data sets.

Table 13: Decompression memory usage of HRCM and other reference-free methods in reference-free compression experiment

Sequence	Decompression memory (KB) by							
	gzip	bzip2	lzma	ppmd	MFC-2	MFC-3	GeCo2	HRCM-S
AeCa	**600**	4,200	9,000	5,600	525,628	2,376,684	525,708	?
AgPh	**600**	4,200	9,000	5,600	525,628	2,376,684	?	?
AnCa	**600**	4,200	9,000	7,648	525,628	2,376,684	525,784	248,328
DaRe	**600**	4,200	9,000	5,600	525,628	2,376,684	2,428,396	255,976
DrMe	**600**	4,200	9,000	5,600	525,628	2,376,684	2,428,400	135,600
EnIn	**600**	4,200	9,000	5,600	525,628	2,376,684	2,393,828	103,268
EsCo	**600**	4,200	9,000	5,600	525,628	2,376,684	525,784	?
GaGa	**600**	4,200	9,000	7,648	525,628	2,376,684	3,442,408	303,912
HaHi	**600**	4,200	9,000	5,600	525,628	2,376,684	525,780	?
HePy	**600**	4,200	9,000	5,600	525,628	2,376,684	525,768	?
HoSa	**600**	4,200	9,000	7,648	525,628	2,376,684	4,001,260	774,308
OrSa	**600**	4,200	9,000	5,600	525,628	2,376,684	2,428,400	135,812
PlFa	**600**	4,200	9,000	5,600	525,628	2,376,684	1,870,060	31,864
ScPo	**600**	4,200	9,000	5,600	525,628	2,376,684	1,869,540	45,296
WaMe	**600**	4,200	9,000	5,600	525,628	2,376,684	525,784	11,616
YeMi	**600**	4,200	9,000	5,600	525,628	2,376,684	?	?
ce6	**704**	4,128	9,048	9,940	525,668	2,376,732	525,784	75,028
TAIR9	**708**	4,128	9,052	7,748	525,672	2,392,408	525,784	50,784
TIGR5.0	**704**	4,128	9,048	7,764	525,680	2,376,720	525,784	116,244
sacCer2	**712**	4,128	3,904	5,684	525,680	2,376,716	525,784	?
Maximum	**712**	4,200	9,052	9,940	525,680	2,392,408	4,001,260	774,308

Bold indicates the best value of the case.

"?" indicates that the decompression is too fast to record the memory usage.

Maximum means the peak memory usage of this method when decompressing the data sets.

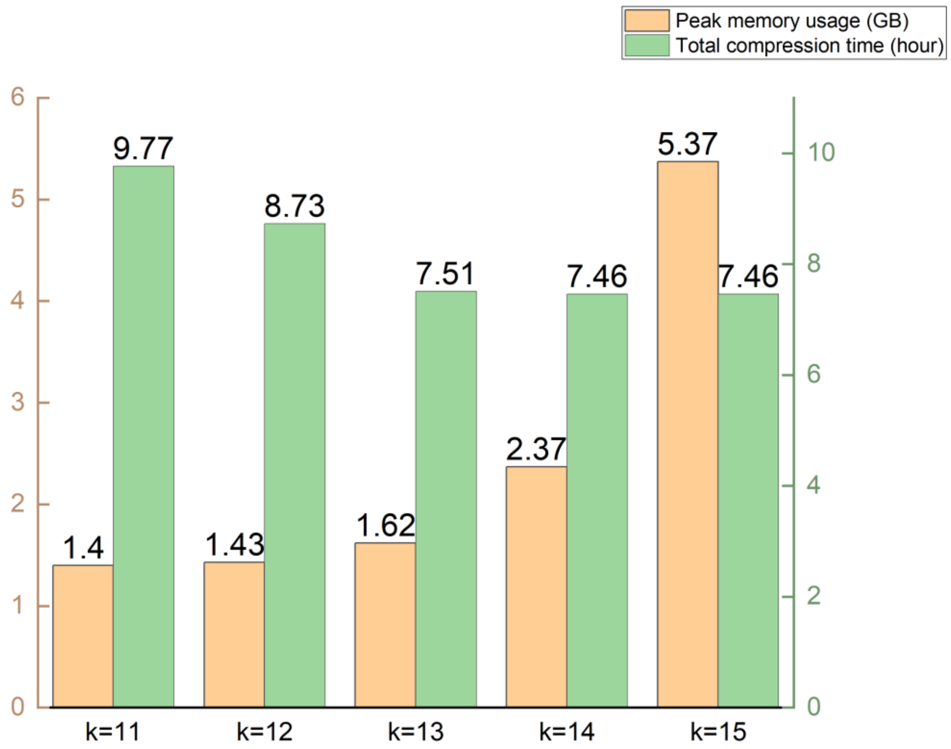

Figure 1: Peak memory usage and total compression time under different values of *k* on the eight benchmark human genomes

4 References

[1] S. Deorowicz, A. Danek, and M. Niemiec, "GDC 2: Compression of large collections of genomes," *Sci Rep*, vol. 5, p. 11565, Jun 25 2015.

[2] D. Pratas and A. J. Pinho, "A DNA Sequence Corpus for Compression Benchmark," *Practical Applications of Computational Biology and Bioinformatics,* vol. 803, pp. 208-215, 2019.
